# HIV-1 Activates Macrophages Independent of Toll-Like Receptors

**DOI:** 10.1371/journal.pone.0003664

**Published:** 2008-12-02

**Authors:** Joseph N. Brown, James J. Kohler, Carter R. Coberley, John W. Sleasman, Maureen M. Goodenow

**Affiliations:** 1 Division of Rheumatology, Immunology and Infectious Diseases, Departments of Pathology, Immunology, and Laboratory Medicine, and Pediatrics, University of Florida College of Medicine, Gainesville, Florida, United States of America; 2 Division of Allergy, Immunology, and Rheumatology, Department of Pediatrics, University of South Florida College of Medicine and All Children's Hospital, St. Petersburg, Florida, United States of America; The Rockefeller University, United States of America

## Abstract

**Background:**

Macrophages provide an interface between innate and adaptive immunity and are important long-lived reservoirs for Human Immunodeficiency Virus Type-1 (HIV-1). Multiple genetic networks involved in regulating signal transduction cascades and immune responses in macrophages are coordinately modulated by HIV-1 infection.

**Methodology/Principal Findings:**

To evaluate complex interrelated processes and to assemble an integrated view of activated signaling networks, a systems biology strategy was applied to genomic and proteomic responses by primary human macrophages over the course of HIV-1 infection. Macrophage responses, including cell cycle, calcium, apoptosis, mitogen-activated protein kinases (MAPK), and cytokines/chemokines, to HIV-1 were temporally regulated, in the absence of cell proliferation. In contrast, Toll-like receptor (TLR) pathways remained unaltered by HIV-1, although TLRs 3, 4, 7, and 8 were expressed and responded to ligand stimulation in macrophages. HIV-1 failed to activate phosphorylation of IRAK-1 or IRF-3, modulate intracellular protein levels of Mx1, an interferon-stimulated gene, or stimulate secretion of TNF, IL-1β, or IL-6. Activation of pathways other than TLR was inadequate to stimulate, via cross-talk mechanisms through molecular hubs, the production of proinflammatory cytokines typical of a TLR response. HIV-1 sensitized macrophage responses to TLR ligands, and the magnitude of viral priming was related to virus replication.

**Conclusions/Significance:**

HIV-1 induced a primed, proinflammatory state, M1_HIV_, which increased the responsiveness of macrophages to TLR ligands. HIV-1 might passively evade pattern recognition, actively inhibit or suppress recognition and signaling, or require dynamic interactions between macrophages and other cells, such as lymphocytes or endothelial cells. HIV-1 evasion of TLR recognition and simultaneous priming of macrophages may represent a strategy for viral survival, contribute to immune pathogenesis, and provide important targets for therapeutic approaches.

## Introduction

Tissue macrophages play a central role in immune response pathways and are major targets for chronic infection by viruses including HIV-1. Infected macrophages constitute a stable viral reservoir that facilitate spread of HIV-1 to other cells or tissue compartments and contribute to immune pathogenesis [1]–[7].

HIV-1 interacts through multiple signaling pathways to reprogram the transcriptome and the proteome of host cells [5], [8]–[13]. HIV-1-induced stimulation of macrophages is a multi-step process that occurs directly in infected cells, as well as indirectly in bystander cells through paracrine signaling. For example, interactions between Env gp120 and the cellular CD4/coreceptor complexes initiate response cascades through intracellular phosphorylation within the MAPK pathway leading to production of tumor necrosis factor (TNF) [10], [14]. While Env gp120 activates multiple signaling pathways, including G-protein coupled receptors (GPCR) [15], protein tyrosine kinase 2 (PYK2) [9], [16], calcium [9], [11], or signal transducer and activator of transcription (STAT) factors [12], other viral proteins also mediate effects on macrophage gene expression. The accessory protein Nef interacts with and inhibits the function of the cellular proapoptotic protein, ASK1 [17]. In addition, HIV-1 genomic RNA activates plasmacytoid dendritic cells (pDC) and monocytes through pattern recognition receptors, specifically Toll-like receptor-7 (TLR7), which initiate a signal transduction cascade to mediate release of TNF and interferons [18], [19] that may contribute to viral replication [20].

Coordinate induction of multiple pathways can lead to cross-talk through convergence of signals at the level of phosphorylation and/or other post-translational modifications resulting in complex cellular responses, such as dynamic cytokine profiles. Convergence of signals is mediated by molecular hubs that include biochemically diverse proteins, such as transcription factors (i.e. NF-κB), kinases (i.e. NEMO or PRKCG), or structural proteins (i.e. YWHAE) [21]–[25]. Molecular hubs integrate pathways into complex networks, regulate the overall organization of signaling networks, amplify or inhibit signals, and increase the complexity of the transcriptional response machinery [26], [27]. Network interactions can be mapped by using systems biology strategies that, in contrast to a single pathway approach, integrate multifaceted analyses to provide a global and dynamic perspective of cellular responses [28], [29].

Macrophage activation is classically defined as M1 (pro-inflammatory) or M2 (regulatory) [30]. Classical macrophage activation involves two steps, priming (e.g. IFN-γ treatment) and triggering (e.g. LPS treatment), which modulate gene or protein expression [31], [32]. In the present study, we used a systems biology approach to integrate genomic and proteomic data across signaling networks with a focus on calcium, apoptosis, and MAPK known from independent pathway studies to respond to viral proteins and contribute to HIV-1 infection [9], [33], [34]. Pathway analysis also incorporated cell cycle and TLR pathways to characterize global and comprehensive host cell response and to define the activation phenotype of macrophages in response to replicating virus.

## Results

### HIV-1 treatment of macrophages stimulates a temporal program of gene expression

**Figure 1:**
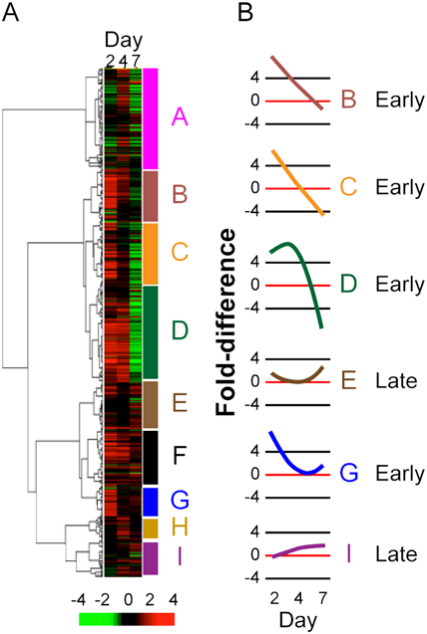
Temporal program of gene expression modulated in HIV-1-treated macrophages. (A) Gene expression values from HIV-1-treated macrophage cultures were divided by expression values from mock-treated cultures to derive fold-change ratios. Median values were calculated for all four donors and hierarchical agglomerative clustering with absolute correlation (un-centered) of approximately 900 genes was based on expression patterns over time. Median increase (red) or decrease (green) of expression ranged between +4 and −4 for each gene. Genes were distributed into nine cluster patterns designated A-I (color coded on right Y axis of the dendrogram) Major branches in the dendrogram were defined by correlation coefficients of greater than 0.75 (mean 0.85, range 0.75 to 0.96). (B) Six cluster patterns were selected for further analysis based on their unique temporal expression patterns. Two patterns of temporal gene expression were observed: early day 2 (clusters B, C, D, and G), and late day 7 (clusters E and I).

Following HIV-1 treatment of macrophages, a subset of 939 probe sets, representing 860 genes that displayed at least 5-fold altered gene expression relative to mock cultures, was analyzed by hierarchical agglomerative clustering (Fig. 1A). Probe sets were distributed into nine major clusters, based on similar patterns of signal intensity values, with about 70% distributed among six clusters. Median temporal change in signal intensity within each of the six clusters was represented graphically as waveforms that fell into two general patterns (Fig. 1B). Relatively higher steady state levels occurred early by day 2 for a majority of the probe sets in clusters B, C, D, and G, while response was delayed in Clusters E and I (Fig. 1B). Overall a majority of genes and proteins were increased in steady-state levels in response to virus. Temporal transcriptome profiles revealed the magnitude, as well as the dynamic nature of macrophage gene expression stimulated by HIV-1 treatment.

The probe sets included genes that were distributed among 67 pathways (data not shown). Altered expression of genes and proteins were organized into the framework of functional pathways. To analyze the pathway networks that reflect classical activation, we focused on five pathways, including calcium, apoptosis, and MAPK, that responded to virus with statistical significance in Pathway Express (*P* = 1.3× 10^−3^, 1.4× 10^−2^, or 6.6× 10^−5^, respectively), as well as cell proliferation and TLR, that apparently failed to respond to virus (*P* = 0.69 or 0.23, respectively).

### Calcium signaling pathway impacted by virus

**Figure 2:**
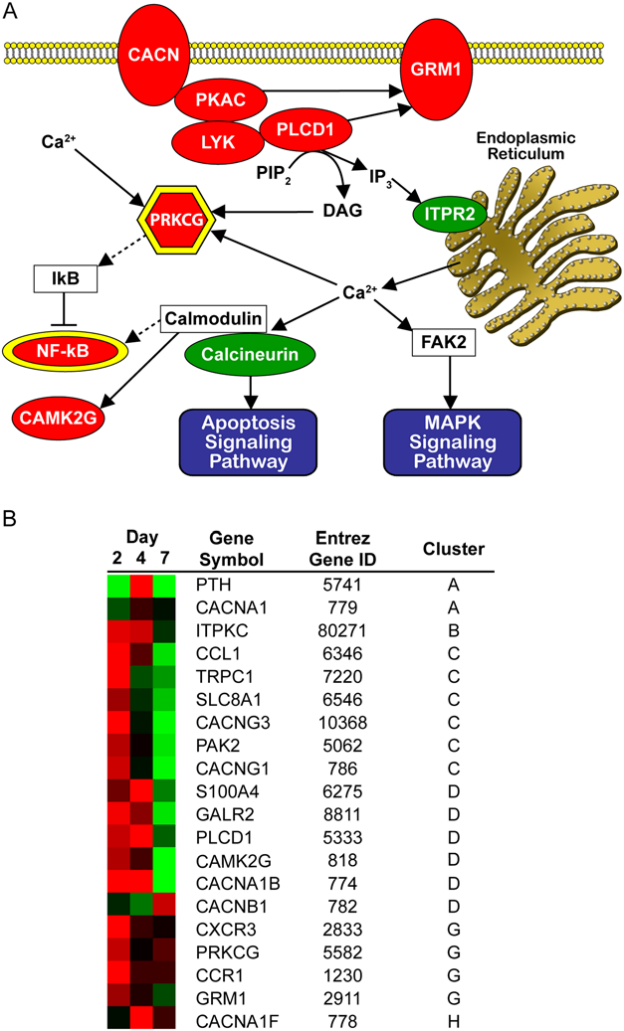
HIV-1 directed effects on calcium release pathways. (A) Model of calcium signaling pathway altered in macrophages by 2 days of HIV-1 treatment. Factors increased in expression by HIV-1 treatment are red and factors expressed at lower levels by virus are green. Ovals represent factors detected at the RNA level and hexagons are factors detected at both the RNA and protein level. Molecular signaling hubs are depicted with a yellow border. (B) Heat map displaying temporal expression of genes whose protein products are involved in calcium signaling over 7 days of HIV-1 treatment relative to mock. The heat map includes Entrez Gene identification number and expression cluster corresponding to the gene. Red indicates expression levels above the mean, black are equal to the mean, and green represents values below the level of the mean.

Calcium signaling is a hallmark of host cell activation and an immediate response to viral recognition [35]. HIV-1 is known to mobilize calcium release from both intracellular and extracellular reservoirs leading to apoptosis and MAPK signaling pathways through molecular hubs [36]–[38]. Collective analyses of our data identified multiple cellular factors comprising calcium-dependent signaling cascades, including the molecular hubs *PRKCG* (Entrez Gene ID: 5582) and *NF-*κ*B* (Entrez Gene ID: 4791) (Fig. 2A). Virus treatment detectably altered 33 (∼15%) of the 266 probe sets associated with calcium signaling. While probe sets were distributed among at least five different expression clusters (A, B, C, D, G, and H), most displayed increased gene expression by day 2 of virus treatment (Fig. 2B).

### Dynamic modulation of apoptotic signaling pathways in HIV-1-treated macrophages

**Figure 3:**
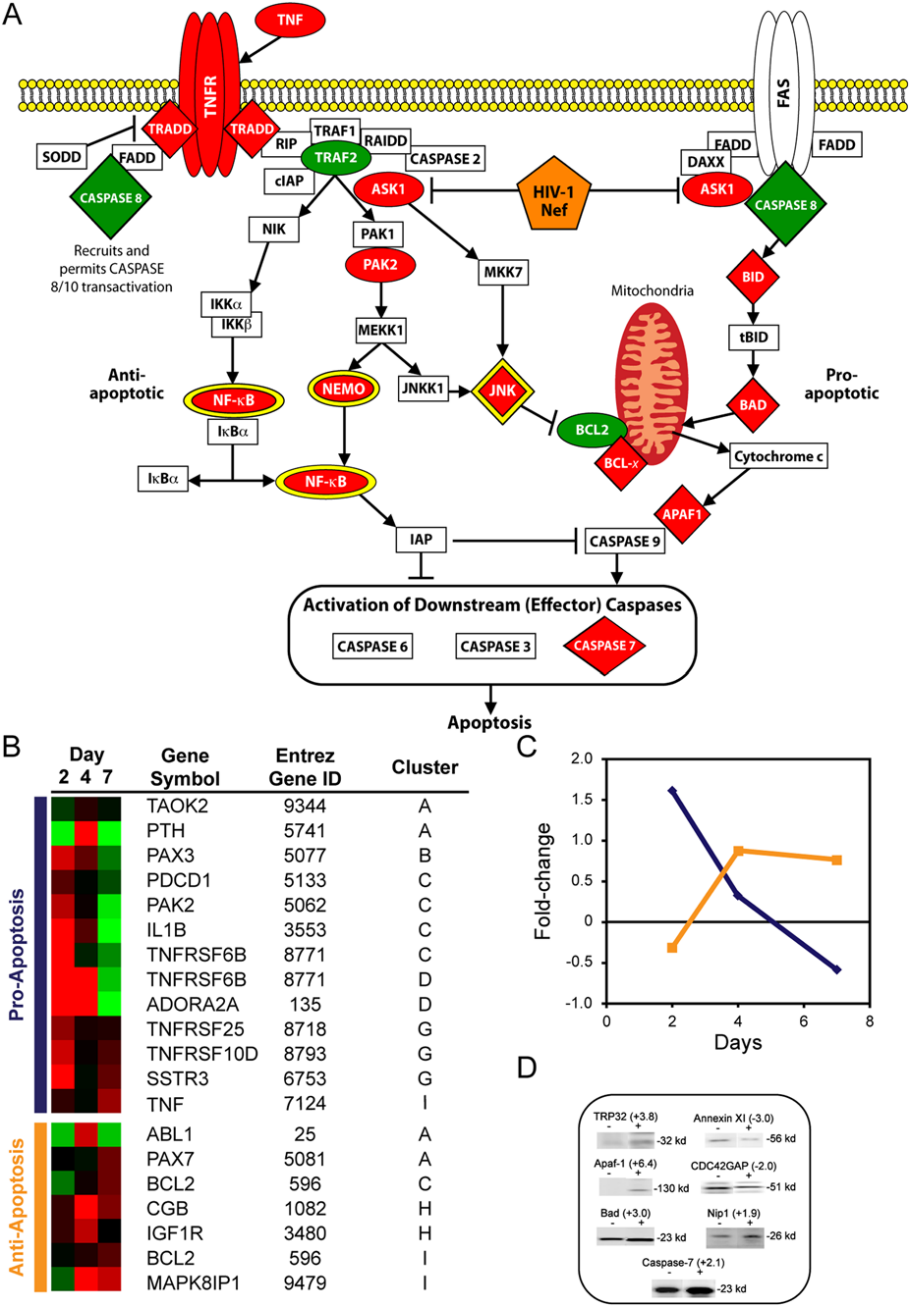
HIV-1 treatment affects expression patterns of members of the apoptotic pathway in primary macrophages. (A) Diagram of the apoptotic signaling pathway depicting factors altered in expression within macrophages upon treatment with HIV-1 within 2 days. Factors increased in expression by HIV-1 treatment are red and factors expressed at lower levels by virus are green. Ovals represent factors altered at the RNA level and diamonds are factors modulated at the protein level. Signaling hubs are depicted with a yellow border. The orange pentagon represents virus-encoded Nef. (B) Heat map representing expression of genes whose protein products are involved in apoptosis. The heat map includes the Entrez Gene identification number and the expression cluster that the gene belongs to. Red indicates expression profiles above the mean, black are equal to the mean, and green represents values below the level of the mean. (C) Median Log_2_ differences of pro-apoptotic genes (blue) and anti-apoptotic genes (orange) in the HIV-treated samples versus mock plotted over 7 days. (D) PowerBlot of apoptotic proteins altered in macrophages by HIV-1 treatment. The signal intensity ratio of HIV-treated to mock-treated is indicated for each protein above the blot.

The apoptosis signaling pathway is composed of two opposing arms, antiapoptosis and pro-apoptosis (Fig. 3A). In contrast to lymphocytes, macrophages exposed to HIV-1 are relatively resistant to apoptosis [39], [40], which led us to expect that macrophages would display an anti-apoptotic transcriptome and proteome response to virus. Surprisingly, within 2 days, when the greatest response to virus occurred, multiple pro-apoptotic factors, including BID (Swiss-Prot ID: P55957), *PAK2* (Entrez Gene ID: 5062), and *ASK1* (Entrez Gene ID: 4217), displayed increased expression, while anti-apoptotic factors, such as *BCL2* (Entrez Gene ID: 596), were decreased in expression (Fig. 3B). Although relative levels of *NF-*κ*B* and *NEMO* (Entrez Gene ID: 8517) increased in response to virus, both are molecular hubs that serve as signaling partners in other pathways, including MAPK and TLRs. Over the course of viral replication, relative expression of pro-apoptotic genes declined and anti-apoptotic genes increased (Fig. 3C). The transcriptome response was extended by analysis of the intracellular proteome compartment that also displayed increased levels of pro-apoptotic proteins, such as BAD (Swiss-Prot ID: Q92934), APAF-1 (Swiss-Prot ID: O14727), and Caspase-7 (Swiss-Prot ID: P55210), within 2 days of viral treatment, while anti-apoptotic proteins, such as CDC42GAP (Swiss-Prot ID: Q07960), showed lower expression levels on day 2 compared to mock (Fig. 3D). Results indicate the dynamic temporal response by macrophages to HIV-1.

### HIV-1 activates the MAPK pathway

**Figure 4:**
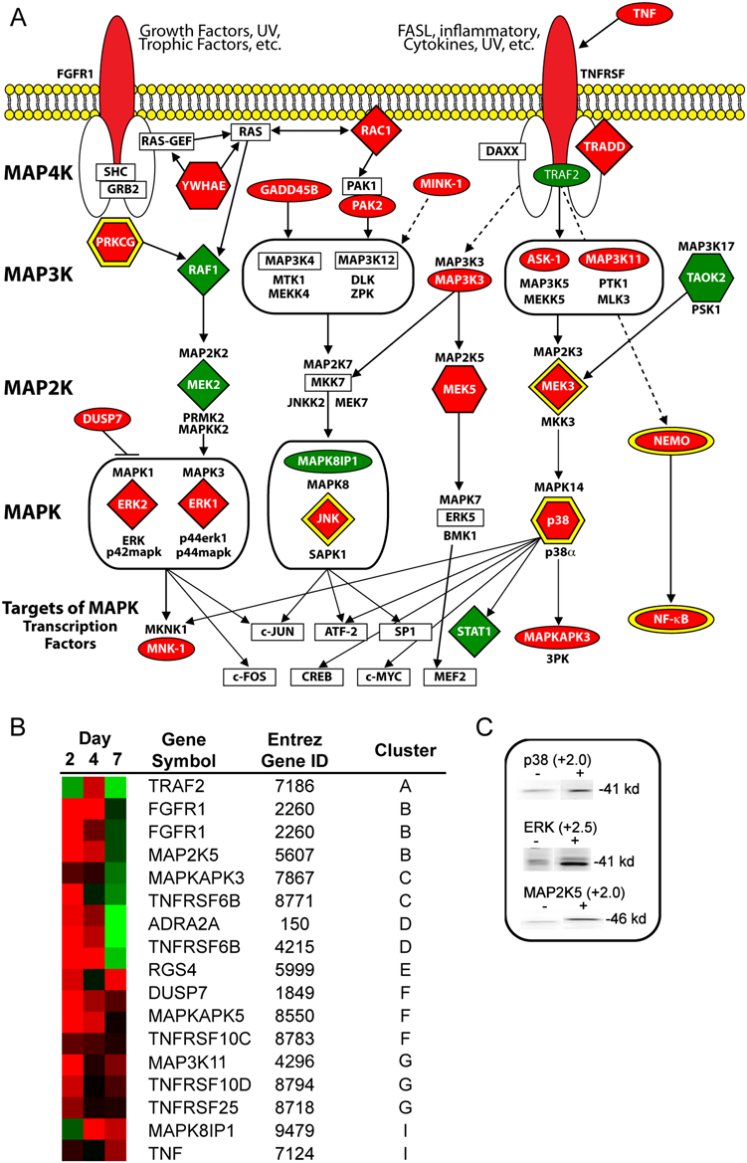
Factors of MAPK pathway altered in expression by HIV-1. (A) Diagram describing MAPK pathway including altered genes and proteins following 2 days HIV-1 treatment. Factors increased in expression by HIV-1 treatment are red and factors expressed at lower levels by virus are green. Ovals represent factors altered at the RNA level, diamonds are factors modulated at the protein level, and hexagons are factors altered at both RNA and protein levels. Signaling hubs are depicted with a yellow border. (B) Expression profiles of genes involved in MAPK signaling and altered by virus treatment over 7 days are displayed in a heat map. Heat map includes Entrez Gene identification number and the expression cluster that the gene belongs to. Red indicates expression profiles above the mean, black equal to the mean, and green represents values below the level of the mean. (C) PowerBlot of MAPK proteins altered in macrophages by HIV-1 treatment. The signal intensity ratio of HIV-treated to mock-treated for each protein is indicated above the blot.

MAPK activation is triggered in response to soluble Env gp120 engagement with surface receptor complex and is required for efficient production of infectious viral particles in primary T-lymphocytes and monocyte/macrophages [41]–[43]. In our studies, virus treatment of macrophages had a global effect on MAPK signaling that extended from cell-surface receptors to transcription factors (Fig. 4A). From a global perspective, HIV-1 had an overall inductive effect on the relative steady state levels of mRNA and proteins involved in the MAPK signaling cascade in macrophages (Fig. 4B and 4C).

### Virus alters macrophage molecular program independent of cell proliferation

Calcium, apoptosis, and MAPK pathways interface through molecular hubs, such as NF-κB, NEMO, PRKCG, JNK (Swiss-Prot ID: P45983), and YWHAE (Swiss-Prot ID: P62258), with multiple signaling pathways, including cell proliferation and TLR. Yet, in contrast to calcium, apoptosis, and MAPK pathways, neither cell proliferation nor TLR pathways was identified as significantly altered by Pathway-Express analysis.

**Figure 5:**
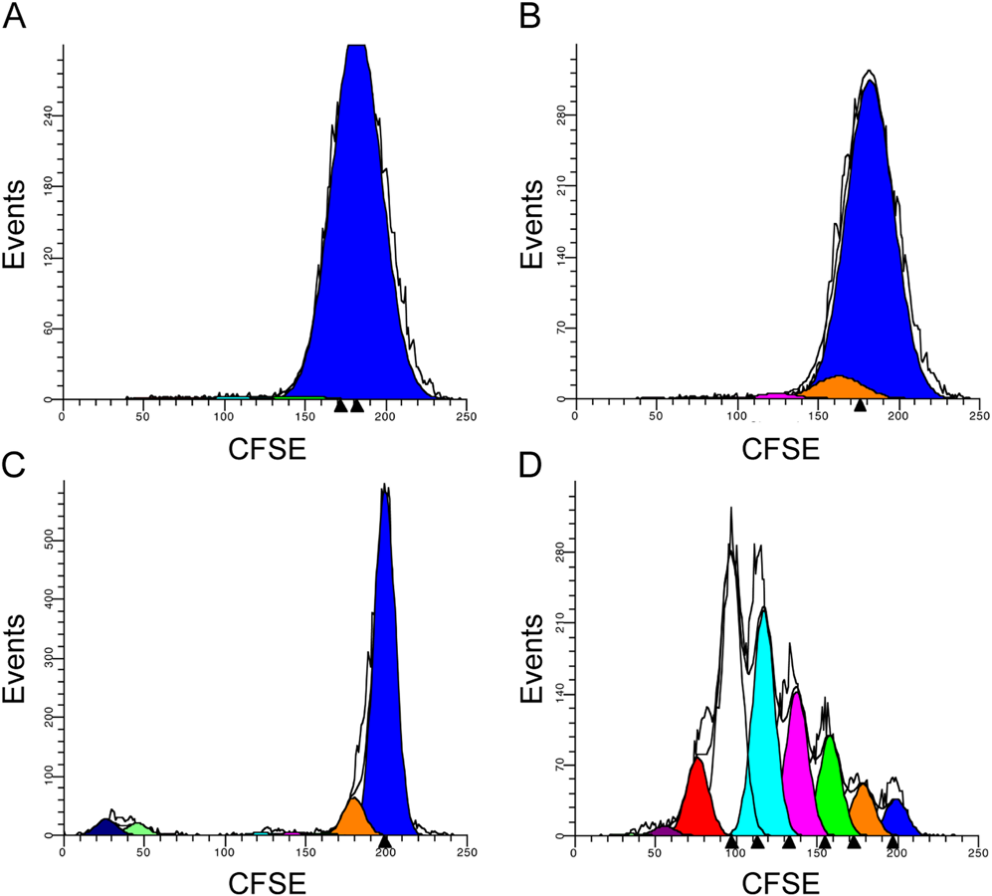
Macrophage activation by virus is independent of cell proliferation. Macrophage cellular proliferation over 10 days (A) in the absence or (B) presence of HIV-1. Cellular proliferation of PBMC in 4 days (C) in the absence of stimulation or (D) the presence of PHA.

Although HIV-1 is capable of crossing the nuclear envelope of terminally differentiated cells without a requirement for proliferation [44], [45], some groups report a portion of macrophages proliferating *in vitro* and have an enhanced susceptibility to HIV-1 infection [46], [47]. In contrast, other groups find that HIV-1 treatment increases the expression of CDK inhibitors, specifically CDKN1A (also known as p21^Cip1/WAF1^, Swiss-Prot ID: P38936), and other targets in the cell cycle important for efficient viral replication [13], [48]. To determine if HIV-1 interactions with macrophages are accompanied by macrophage proliferation in our studies, two approaches were used. HIV-1- or mock-treated macrophages labeled with propidium iodide showed no differences in DNA content over 10 days (data not shown). A more sensitive assessment of proliferation involved labeling cellular proteins with the fluorescent molecule, CFSE [49]. When CFSE-labeled macrophages were cultured for 7 days in the presence or absence of HIV-1 no change in fluorescent intensity was detected (Fig.5A–B). Other treatments, such as IL-3 and GM-CSF, also failed to induce proliferation of macrophages (data not shown). For comparison, cellular proliferation in PBMC was evaluated. A single peak of fluorescence in unstimulated PBMC after 4 days indicated a quiescent population of cells (Fig. 5C), while PHA stimulation produced six discrete populations with fluorescence intensities less than that of unstimulated PBMC reflecting cell proliferation among more than 96% of PBMC (Fig. 5D). Taken together, results demonstrate that virus-related modulation of the macrophage transcriptome and proteome was independent of proliferation.

### Coordinate stimulation of macrophage transcriptome is independent of TLR signaling

**Figure 6:**
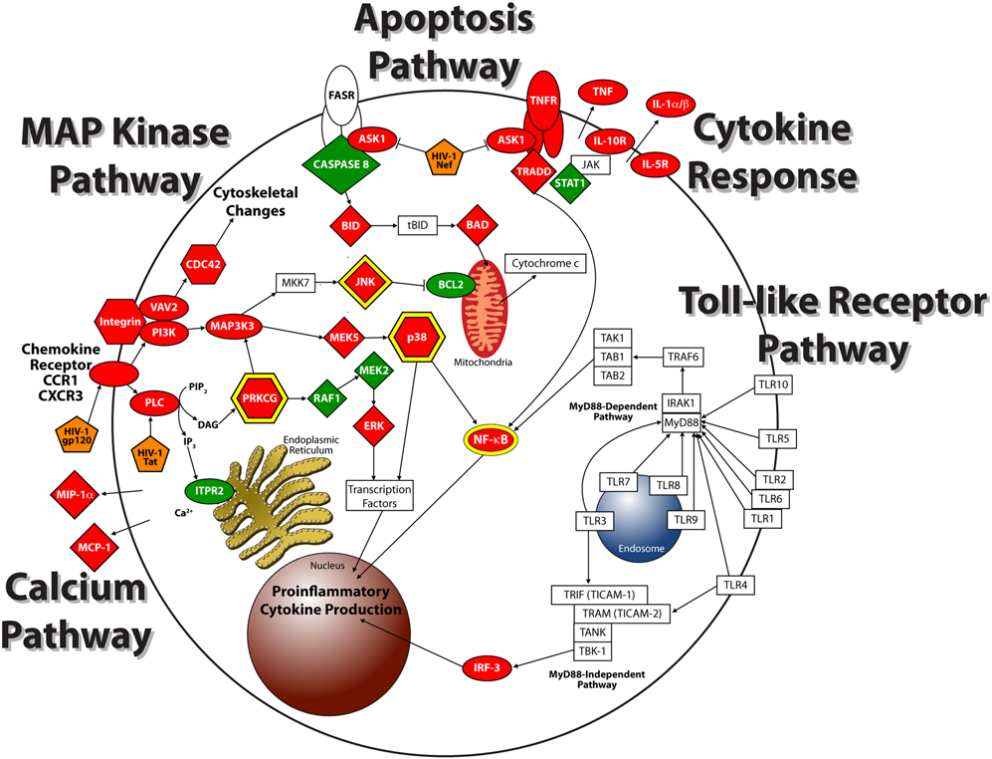
Summary of signaling pathways altered or evaded by virus. Factors expressed at higher levels in HIV-treated than mock are red and factors expressed at lower levels in HIV-treated than mock are green. Ovals represent factors detected at the RNA level, diamonds are factors detected at the protein level, and hexagons are factors detected at both RNA and protein. Signaling hubs are depicted with a yellow border.

**Figure 7:**
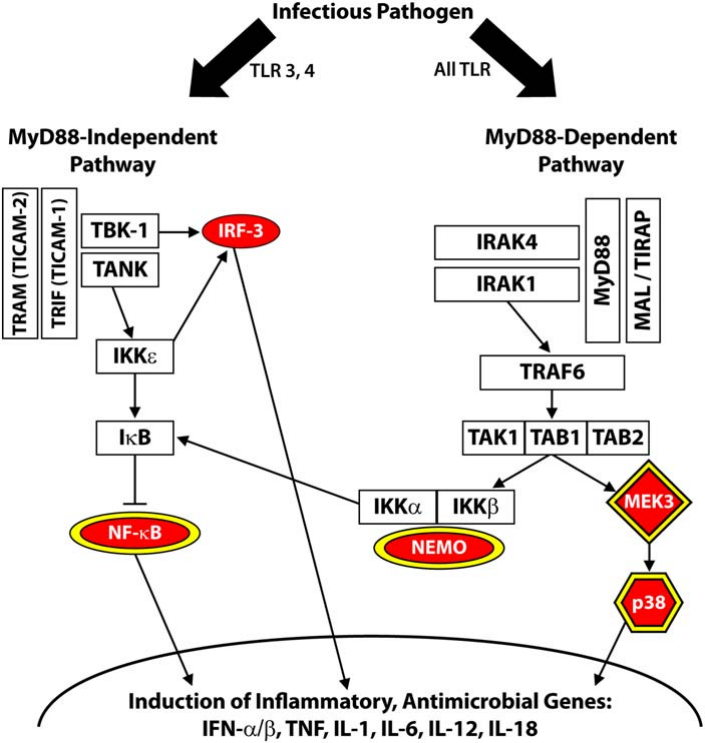
TLR pathway is unaltered by HIV-1. TLR signaling pathway. Factors increased in expression by HIV-1 treatment are red and factors expressed at lower levels by virus are green. Ovals represent factors detected at the RNA level. Signaling hubs are depicted with a yellow border.

Activation across calcium, apoptosis, and MAPK pathways converge on NF-κB, a transcription factor that functions as a molecular hub (Fig. 6). Yet, not all pathways leading to or interfacing with NF-κB responded to virus in our studies. A striking example was the TLR pathway that displayed essentially no response over the course of viral replication. Although proteins, particularly phosphoproteins, in the TLR pathway were underrepresented in the PowerBlot analysis, transcriptome analysis included 27 members of the TLR signaling pathways (Fig. 7). Only five displayed increased expression. Increased expression of *IRF-3* (Enrez Gene ID: 3661) was detected in only one of three macrophage donors, while *NF-*κ*B*, *NEMO*, MAP2K5 (Swiss-Prot ID: Q13163), and p38 (Swiss-Prot ID: Q16539) function as molecular hubs in multiple signaling pathways and are not restricted to TLR pathways.

### HIV-1 escapes TLR response

**Figure 8:**
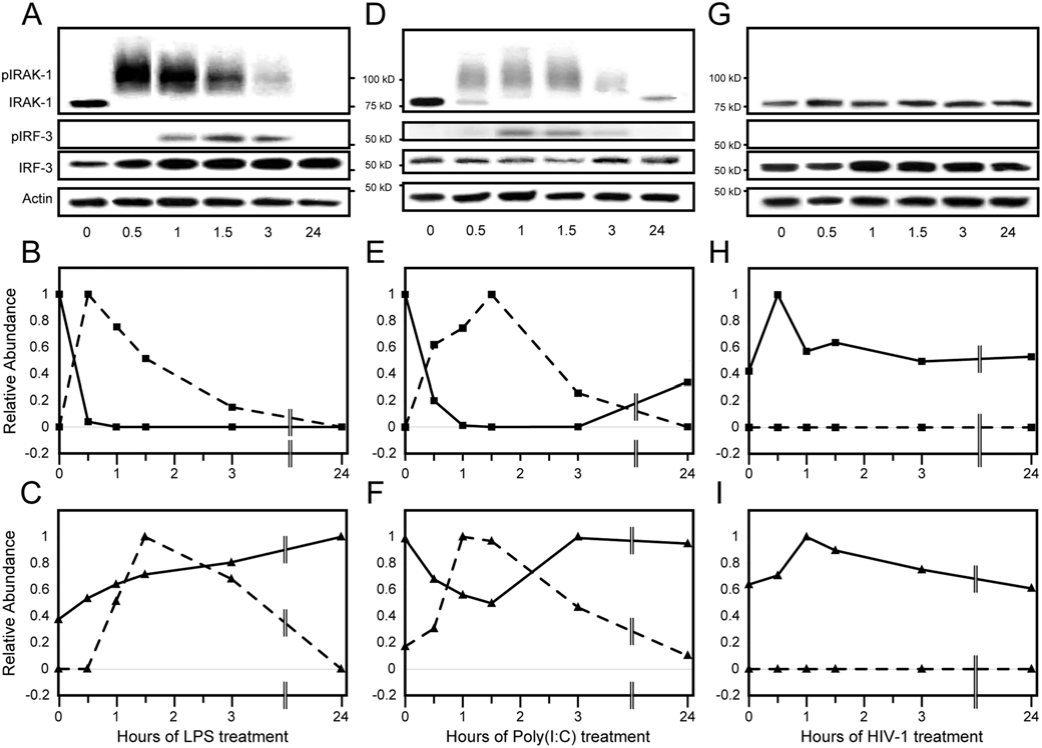
HIV-1 fails to activate TLR in macrophages. Macrophages were treated with LPS (A–C), Poly(I:C) (D–F), or HIV-1 (G–I) for 24 hours. Whole cell lysates from the indicated hours of treatment were western blotted for IRAK-1, p-IRF-3, IRF-3, and actin (A, D, and G). Relative abundance levels of IRAK-1 (solid line, squares) and p-IRAK-1 (dotted line, squares) from western blot are displayed in line graphs (B, E, and H). IRF-3 (solid line, triangles) and p-IRF-3 (dotted line, triangles) relative abundance levels are also represented in line plots (C, F, and I).

TLR signaling is transmitted through a MyD88-dependent and/or, depending on the TLR, a MyD88-independent pathway (Fig. 7). Both pathways result in production of proinflammatory cytokines, such as type I interferons and TNF (Swiss-Prot ID: P01375) [50]. To determine if proximal signaling molecules respond to virus, we evaluated phosphorylation of TLR signaling members, IRAK-1 (specific for the MyD88-dependent pathway, Swiss-Prot ID: P51617) and IRF-3 (member of both pathways). Treatment of macrophages with TLR agonists LPS (primarily TLR4 agonist) or Poly(I:C) (primarily TLR3 agonist) produced phosphorylated forms of IRAK-1 within 30 minutes or IRF-3 within one hour (Fig. 8A–F). Phophorylated forms of IRAK-1 or IRF-3 appeared transiently and decreased by 3 hours. Steady-state levels of total IRF-3 protein remained relatively stable over the course of treatment, while diminished levels of IRAK-1 following LPS treatment failed to recover after 24 hours (Fig. 8B) or 48 hours (data not shown). In contrast to other ligands, HIV-1 treatment of macrophages produced a transient increase in IRAK-1, but no phosphorylation of either IRAK1 or IRF3 (Fig. 8G–I).

**Figure 9:**
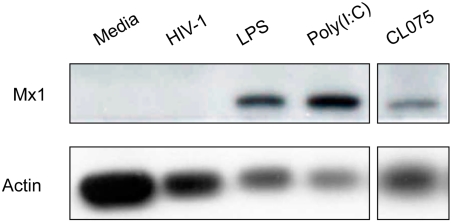
Macrophages fail to produce Mx1 in response to HIV-1. Whole cell lysates derived from macrophages treated with media, HIV-1, LPS, Poly(I:C), or CL075 for 24 hours were immunoblotted for Mx1 or actin protein expression.

The relatively limited proportion of macrophages infected by HIV-1 within the first 24 hours could limit detection by western blot of phosphorylated/activated IRAK-1 or IRF3. To address this issue, levels of Mx1 protein (Swiss-Prot ID: P20591) were measured. Mx1 (Swiss-Prot ID: P20591), an antiviral interferon-stimulated gene, is a relatively stable, sensitive marker for low levels of interferon and innate immune activation [51]. Although treatment of macrophages through multiple TLRs 4, 3, or 8 for 24 hours stimulated increased intracellular levels of the Mx1 protein, treatment by HIV-1 induced no expression of Mx1 protein (Fig. 9). Although macrophages recognize and respond to TLR agonists via MyD88-dependent or independent pathways, HIV-1 has no apparent impact on targets downstream in TLR signaling through either pathway. HIV-1 interactions with macrophages were insufficient to trigger proinflammatory cytokine responses indicative of classical activation.

### HIV-1 primes macrophages for enhanced response to TLR agonists

**Figure 10:**
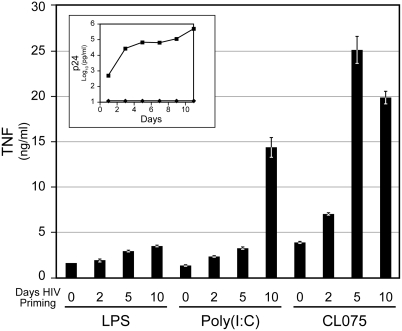
HIV-1 spread correlates with increased TLR response. Macrophages were treated with HIV-1 or media alone for 24 hours. Virus was allowed to spread in culture for an additional 9 days. Viral replication in culture is represented by supernatant p24 levels (inset). Diamonds represent untreated and squares represent HIV-1 treated. At the designated days of HIV-1 priming, cells were treated for 24 hours with LPS, Poly(I:C), or CL075. Supernatants were collected and levels of TNF are represented in the bar graph.

Stimulation of macrophages through TLR signaling rendered cells refractory to HIV-1 infection (data not shown) [52]–[55]. Consequently, to determine if HIV-1 primes macrophages, an initial step in classical activation, a spreading viral infection was established that produced a three log increase in supernatant p24 over 10 days (Fig. 10, inset). On days 2, 5, and 10 post infection, macrophages were stimulated for 24 hours with ligands for TLR 4 (LPS), 3 (Poly(I:C)), and 8 (CL075). Enhanced TNF levels were observed as early as 2 days and increased over time to a maximum between days 5 and 10, paralleling viral infection (Fig. 10).

**Figure 11:**
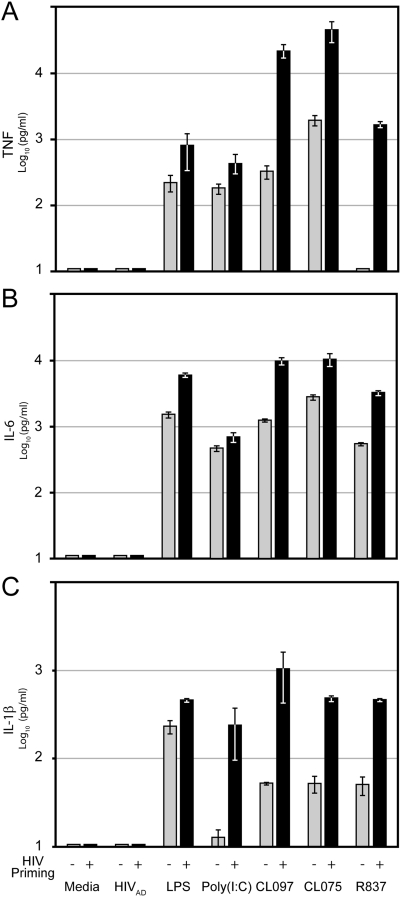
HIV-1 priming enhances macrophage cytokine production in response to TLR stimulation. Macrophages were treated with HIV-1 or media alone for 24 hours. Virus was allowed to spread in culture for an additional 9 days. Macrophages in the absence (gray bars) or presence (black bars) of spreading virus were treated with media, HIV-1, LPS, Poly(I:C), CL097, CL075, or R837 for 24 hours, and supernatant TNF (A), IL-6 (B), or IL-1β (C) levels were measured.

To examine the breadth of the priming response, additional TLR ligands and proinflammatory cytokines, which are amplified as a consequence of innate immune activation, were analyzed at the time of maximum response, day 10. Macrophages were treated for 24 hours with any one of a variety of TLR ligands, including LPS, Poly(I:C), and CL097 (TLR7/8 agonist), as well as CL075 (TLR8 agonist) or R837 (TLR7 agonist) to distinguish differences that might result from selective activation through individual TLRs. Overall, each agonist stimulated production of TNF, IL-1β (Swiss-Prot ID: P01584), and IL-6 (Swiss Prot ID: P05231) (Fig. 11A–C, gray bars). In contrast, no proinflammatory cytokines were detected with HIV-1 treatment alone. Yet, when HIV-1 infected macrophage cultures were treated with TLR agonists, increased levels of cytokines were produced (Fig. 11A–C, black bars). HIV-1 infection of macrophages increased LPS response by about 3- to 5-fold, while response to other TLR ligands was increased by a factor of 10 or greater. Results indicate that HIV-1 can provide an effective priming signal to macrophages, which sensitizes the cells to TLR agonists and enhances proinflammatory cytokine responses.

## Discussion

HIV-1 stimulates dynamic and complex responses within macrophages through alteration of expression or modification of cellular genes or proteins. While virus modulates multiple signaling pathways over the course of infection, HIV-1 has a selective, rather than global, impact on macrophages. Our analysis identified a complex interplay, through molecular hubs, to establish spatial-temporal relationships across signaling pathways that organize virus-specific activation programs into an HIV-1 response phenotype for macrophages.

Pathway analysis provides a rational and unsupervised statistical algorithm for integrating large transcriptome and proteome data sets and quantifying viral impact on the pathways. High-throughput transcriptome discovery across multiple pathways enhanced by proteomic approaches reduces the significance of outlier data points, such as increased IRF-3 gene expression by HIV-1 in a single donor, which may be difficult to interpret on an individual pathway basis by a single method. Our study was designed to discern physiological responses in a population of macrophages exposed to virus, rather than to distinguish between the direct and indirect effects of virus on cells. Yet, even though only a minority of macrophages was infected by HIV-1 within 2 days, the initial round(s) of virus replication produced the greatest differences in gene expression raising the possibility that the macrophage response to HIV-1 involved bystander cells.

Viral impact on signaling is frequently studied by investigating discrete pathways in cells treated with an isolated viral component, for example Env gp120 [9], [11], [56], Tat [57], [58], Nef [34], [59], Vpr [60], or synthetic RNA corresponding to subgenomic regions of HIV-1 [18]. While these studies have provided a framework for network analysis, our global strategy builds insight through the use of whole virus replication to assess the coordinate impact on multiple cell signaling pathways by viral gene expression during spreading infection. Several pathways in macrophages, including calcium, MAPK, and apoptosis, which are impacted by individual viral components, were also modulated at the level of gene and protein expression over the course of viral replication. In contrast, Env gp120 treatment of macrophages induces the production of TNF [10], while our studies, unexpectedly, failed to detect TNF secretion in response to HIV-1, which could reflect differences in signaling between monomeric Env gp120 and virion-associated trimeric Env complexes, quantitative difference between envelope glycoproteins in soluble or virion form, or activity by viral proteins post-entry to suppress signaling. The fact that multiple signaling pathways can result in similar cytokine responses argues that a global analysis of all pathways simultaneously is important in interpreting cellular response.

Changes in macrophage cell cycle or pathways can be critical for productive HIV-1 infection [13], [48]. Our studies demonstrate that changes in cell proliferation pathways by virus are independent of DNA doubling or cell proliferation. Therefore, transitions within compartments of G1, prior to DNA replication during S phase, may be important for establishing an optimal intracellular milieu for viral infection [47], [61]. Alternatively, cell cycle factors may be essential for HIV-1 replication in macrophages independent of their role in cell cycle.

Human macrophages express a repertoire of TLRs with functional responses to natural ligands or agonists. Nonetheless, our studies indicated that TLR recognition of HIV-1 replicating in macrophages is impaired, not only during initial infection, but over the course of viral spread, ruling out the possibility that infection of small numbers of cells limited detection of TLR signaling. Manipulation of TLR signaling by a variety of mechanisms is a survival technique evolved by a number of viruses, including human cytomegalovirus, influenza virus, and hepatitis C virus [22], [62]–[64]. Cytosolic pattern recognition receptors, RIG-I and MDA5, cross-talk through molecular hubs, such as NF-κB and IRF-3, result in proinflammatory cytokine responses similar to responses through TLRs. Our results indicate that HIV-1 infection involves global evasion of molecular pattern recognition receptors within macrophages. HIV-1 might passively evade pattern recognition, actively inhibit or suppress recognition and signaling, or require dynamic interactions between macrophages and other cells, such as lymphocytes or endothelial cells.

Immune activation and inflammatory responses correlate with HIV-1 disease pathogenesis [65], and macrophages are central regulators of systemic immune activation and targets of HIV-1 infection [40], [66]. Macrophage activation is a multistep process resulting in at least two major categories of activation, M1 or classical activation and M2 or alternative activation [30]. Classical macrophage activation involves two steps, priming and triggering, which modulate gene or protein expression [31], [32]. HIV-1 can stimulate macrophages through a variety of signaling pathways, including calcium, MAPK, and apoptosis, which are connected with TLR through the NF-κB molecular hub. Nonetheless, HIV-1 fails to induce TLR-mediated activation indirectly through cross-talk or as a direct trigger. The TLR signaling cascade, which can be activated in pDC by HIV-1 RNA, failed to promote activation in macrophages in response to HIV-1, indicating that viral evasion of TLR may be a cell-type and/or virus-specific survival strategy.

Even though virus was inadequate to provide classical activation of macrophages through TLR signaling, HIV-1 clearly primed macrophages to be hypersensitive to TLR agonists. While the viral doses applied to macrophages in our studies were insufficient to result in detectable levels of TNF, IL-1β, or IL-6 secretion, cells primed by HIV-1 produced enhanced cytokine levels following TLR ligand treatment. HIV-1-induced priming of macrophages is distinct from traditional IFN-γ priming signals, based on STAT1 phosphorylation and multimerization as well as expression of interferon-stimulated genes, in particular Mx1 [12], [67], [68]. The novel activation program induced macrophages by HIV-1 can be designated M1_HIV_. TLR stimulation results in a state of tolerance, due to degradation of activated signaling members within the TLR pathway, such as IRAK1, which prevents cells from mounting a response upon subsequent challenge. M1_HIV_-activated macrophages can undergo TLR tolerance upon stimulation with TLR ligands, indicating viral proteins do not mimic TLR signaling. Macrophage sensitization to microbial components may contribute to disease progression in HIV-1-positive individuals who have compromised gut-associated lymphoid tissues and increased microbial translocation into the plasma [69].

Several possible mechanisms may account for macrophage sensitization to TLR ligands during HIV-1 infection. While activation of pattern recognition receptors, such as TLRs, can induce positive-feedback loops resulting in increased expression of TLRs and proinflammatory cytokines, HIV may selectively activate specific components of this response in macrophages through signaling cross-talk, independent of TLR recognition, resulting in increased TLR expression levels [70] in the absence of proinflammatory cytokine production. For example, activity of NF-κB during HIV infection may contribute to TLR-independent enhanced responsiveness of macrophages. Consequently, novel therapeutics might selectively suppress exaggerated innate immune responses induced by HIV-1 infection, without inhibiting protective TLR responses.

Macrophages regulate HIV-1 disease progression through their secretory products [40], [66]. HIV-1-induced macrophage sensitization may correlate with poor clinical outcome if priming *in vivo* increases disease sequelae by coinfecting pathogens. Viral tropism and coreceptor-specificity change over the course of disease [71], raising the possibility that viruses may evolve in their potential to prime macrophages concomitant with the evolution of coreceptor use from CCR5 to CXCR4 [72], [73] or increased tropism for macrophages by CCR5-using viruses [74]. Macrophage priming potential by quasispecies of HIV-1 may provide a novel biomarker for assessing the course of HIV-1 disease progression within infected individuals.

## Materials and Methods

### Preparation of viral stocks

A stock of macrophage-tropic, CCR5 using HIV-1_JR-FL_ (NIH Aids Reference and Reagent Program, Rockville, MD) was prepared in peripheral blood mononuclear cells (PBMC), as previously described [75]. A stock of macrophage-tropic CCR5 using HIV-1AD was prepared by transfection of the molecular clone into 293T cells [76]. Viral stocks were titered on PBMCs as previously described [75].

### Viral treatment of macrophages

Monocytes from healthy human donors were differentiated into macrophages over the course of 7 days in culture, and treated with either HIV-1_JR-FL_ (15,000 TCID_50_ per ml) or mock supernatants for 24 hours as previously described [13]. Four independent experiments using macrophages from different donors provided a 90% chance of detecting a two-fold difference between virus- and mock-treated cultures, based on analysis of variance using a randomized complete block design, a two-sided t-test, and a standard deviation of 0.5.

Total RNA for each sample was extracted and prepared for hybridization according to GeneChip® for use with HG-U95A (version 2) arrays (Affymetrix, Santa Clara, CA). Specific conditions were described previously [13]. High-resolution chip images were analyzed using Microarray Suite Version 4 (Affymetrix). Signal intensity values from microarrays were normalized and converted to fold-change values as previously described [13]. GeneChip information was submitted to the Gene Expression Omnibus at the NCBI (GEO: GSE13395).

### RNA data analysis

A custom relational database was designed using Microsoft Access 2003® on the Windows XP Pro® platform (Microsoft Corporation, Redmond, WA). The database contains all raw data, as well as links between unique gene identifiers for mRNA and protein, functional categories, and summary of function. Functional categories of genes were based on the Gene Ontology (GO) Consortium public domain database (http://www.geneontology.org/). Cluster analysis was performed to gain an overview of temporal gene expression patterns within the dataset. Prior to clustering, filters were applied so that a robust set of genes could be analyzed. Genes were first filtered to remove any transcripts that were considered absent under all conditions. A subsequent filter identified genes in infected cultures that displayed greater than four-fold change (induced or repressed) in hybridization intensities relative to levels in uninfected cultures. Average linkage hierarchical agglomerative clustering with un-centered correlation was performed on the median values obtained for each probe set for all donors at each time point using the software package Cluster (Mike Eisen, Berkeley, CA). Major branches in the dendrogram were defined by correlation coefficients of greater than 0.75 (mean 0.85, range 0.75 to 0.96). Probe sets were classified as altered in expression, expressed exclusively in HIV-1-treated, or mock-treated macrophages, as previously described [13].

### Intracellular protein determinations

Total proteins were extracted from macrophages from each of four donors following treatment as previously described [13]. Macrophages from two of the four donors were assayed for both proteins and mRNA. Immunoblot analysis of about 1000 intracellular proteins was performed by BD Biosciences (BD Biosciences, Franklin Lakes, NJ). Approximately 860 proteins, including 33 phosphorylated targets, were identified by an Entrez Gene ID. The Affymetrix GeneChips contained probe sets that matched 567 proteins assayed for in the PowerBlot. Proteins were considered altered by virus- relative to mock-treatment if expression differed ≥1.5 fold, corresponding to 305 proteins, and 18 of these proteins were also altered at the gene level.

For immunoblot analysis of phosphoproteins in the TLR pathway, countercurrent centrifugal elutriated monocytes were kindly provided by Howard Gendelman and differentiated into macrophages over 7 days at 37°C and 5% CO2 in the presence of 10% heat-inactivated pooled human serum, 1% glutamine, 10 µg/ml ciprofloxacin, and 1000 U/ml recombinant MCSF [77]. Cells (3×10^6^ cells per well) were treated for indicated time periods with 1 mg/ml E.coli 0111:B4 LPS (Sigma-Aldrich, St. Louis, MO), 100 mg/ml Poly(I:C) (Invivogen, San Diego, CA), or 15,000 TCID_50_ HIV-1_AD_. Following treatment, macrophages were washed twice with phosphate-buffered saline (PBS), pH 7.4, and immediately lysed in ice-cold lysis buffer (1% Triton X-100, 2 mM Tris-HCl, 150 mM NaCl, 5 mM EDTA, 1 mM DTT, 1 mM NaF, 1mM Sodium Orthovanadate, 1 Protease Inhibitor Cocktail Tablet (Roche, Basel, Switzerland), 1% Phosphatase Inhibitor Cocktail I (Sigma-Aldrich), and 1% Phosphatase Inhibitor Cocktail II (Sigma-Aldrich)). Whole-cell lysates were centrifuged at 6,000× g for 10 min at 4°C, and the supernatants were frozen at −80°C. The protein concentrations of cell extracts were determined by the BCA protein assay (Bio-Rad, Hercules, CA). Aliquots of cell extracts containing 15 µg of total proteins were diluted in Laemlli Sample Buffer with 5% β- mercaptoethanol, and resolved on 4–20% sodium dodecyl sulfate-polyacrylamide gel electrophoresis (SDS-PAGE) and transferred to a PVDF membrane (Bio-Rad). Membranes were blocked with 5% milk in TTBS (10 mM Tris, pH 7.4, 100 mM NaCl, 0.1% Tween 20) for 1 hour at room temperature and probed with mouse anti-IRAK-1 antibody (Santa Cruz Biotechnology, Santa Cruz, CA), rabbit anti-pIRF-3 (Cell Signaling Technology, Danvers, MA), mouse anti-IRF-3 (Santa Cruz Biotechnology), goat anti-Mx1 (Santa Cruz Biotechnology), or mouse anti-β-actin (Santa Cruz Biotechnology), followed by goat anti-mouse IgG-HRP (Cell Signaling Technology), mouse anti-goat IgG-HRP (Santa Cruz Biotechnology), or goat anti-rabbit IgG-HRP (Cell Signaling Technology). Horseradish peroxidase activity was visualized through enhanced chemiluminescence detection Visualizer (Upstate, Charlottesville, VA) for low abundant proteins, such as p-IRF-3, or Luminol (Santa Cruz Biotechnology) followed by exposure to CL-Xposure film (Thermo Scientific, Waltham, MA). Relative abundance of bands was measured in Adoby Photoshop CS3 (San Jose, CA). Density of individual bands were normalized to respective actin for each lane.

### Secreted cytokine determination

Elutriated monocytes from three independent donors were plated at a density of 2.5×10^5^ cells per well in a 48 well plate, and differentiated for 7 days into macrophages as previously described. Macrophages were treated with either media or 5,000 TCID_50_ HIV-1_AD_ (MOI = 0.02) for 24 hours. Cells were washed with PBS and incubated in media for 9 days with 50% media changes every 2 days. Macrophages were washed with PBS and treated for 24 hours with 1 µg/ml *E.coli* 0111:B4 LPS (Sigma-Aldrich), 100 µg/ml Poly(I:C) (Invivogen), 1 µg/ml CL097 (Invivogen), 1 µg/ml CL075 (Invivogen), 10 µg/ml R837 (Invivogen), 5,000 TCID_50_ HIV-1_AD_, or media. Supernatants were collected and levels of TNF, IL-6, and IL-1β were quantified by ELISA (BD Biosciences).

### Proliferation assay

Freshly isolated PBMCs were purified and washed as previously described [75]. Macrophages and PBMCs were treated at room temperature for 8 minutes with 5 µM CFDA-SE (Invitrogen, Carlsbad, CA). The reaction was immediately quenched with the addition of PBS plus 10% FBS and cells were washed 5 times with 1× PBS. Macrophages were incubated with either media or media plus 50,000 TCID_50_ HIV-1_AD_ (MOI = 0.017) for 24 hours at 37°C. Macrophages were washed once with 1× PBS and incubated in media for 9 days at 37°C with 50% media changes every 2 days. After 10 days in culture with either media or virus, macrophages were washed 4 times with 1× PBS and gently scrapped. PBMCs were isolated and cultured as previously described [78]. Cells were treated with media or 9 mg/ml PHA (Sigma-Aldrich) for 4 days with 50% media changes every 2 days, and washed 4 times with 1× PBS. Cells were pelleted through centrifugation at 250× *g* for 10 minutes, resuspended in 1 ml 1× PBS, and fluorescent intensity was measured by flow cytometry in a BD FACSCalibur (BD Biosciences).

### Pathway analysis

Pathway-Express [43], was used to identify signaling pathways in macrophages altered with statistical significance [*P*<0.05]. Signaling pathways were constructed from several sources, including Kyoto Encyclopedia of Genes and Genomes (KEGG) (Kanehisa Laboratory, Bioinformatics Center, Institute for Chemical Research, Kyoto University), BioCarta, Inc. (San Diego, CA), Protein Lounge (San Diego, CA), and literature searches. Lists of genes and proteins whose relative expression level differences met the criteria as altered by viral treatment were integrated and displayed on constructed signaling pathways. Factors that participate in more than one signaling pathway were considered molecular hubs.

## References

[1] Khati M, James W, Gordon S (2001). HIV-macrophage interactions at the cellular and molecular level.. Arch Immunol Ther Exp (Warsz).

[2] Fischer-Smith T, Croul S, Sverstiuk AE, Capini C, L'Heureux D (2001). CNS invasion by CD14+/CD16+ peripheral blood-derived monocytes in HIV dementia: perivascular accumulation and reservoir of HIV infection.. J Neurovirol.

[3] Ho WZ, Cherukuri R, Douglas SD (1994). The macrophage and HIV-1.. Immunol Ser.

[4] Schuitemaker H (1994). Macrophage-tropic HIV-1 variants: initiators of infection and AIDS pathogenesis?. J Leukoc Biol.

[5] Del Corno M, Liu QH, Schols D, de Clercq E, Gessani S (2001). HIV-1 gp120 and chemokine activation of Pyk2 and mitogen-activated protein kinases in primary macrophages mediated by calcium-dependent, pertussis toxin-insensitive chemokine receptor signaling.. Blood.

[6] Williams KC, Corey S, Westmoreland SV, Pauley D, Knight H (2001). Perivascular macrophages are the primary cell type productively infected by simian immunodeficiency virus in the brains of macaques: implications for the neuropathogenesis of AIDS.. J Exp Med.

[7] Kim WK, Avarez X, Williams K (2005). The role of monocytes and perivascular macrophages in HIV and SIV neuropathogenesis: information from non-human primate models.. Neurotox Res.

[8] Giri MS, Nebozhyn M, Showe L, Montaner LJ (2006). Microarray data on gene modulation by HIV-1 in immune cells: 2000–2006.. J Leukoc Biol.

[9] Lee C, Liu QH, Tomkowicz B, Yi Y, Freedman BD (2003). Macrophage activation through CCR5- and CXCR4-mediated gp120-elicited signaling pathways.. J Leukoc Biol.

[10] Lee C, Tomkowicz B, Freedman BD, Collman RG (2005). HIV-1 gp120-induced TNF-{alpha} production by primary human macrophages is mediated by phosphatidylinositol-3 (PI-3) kinase and mitogen-activated protein (MAP) kinase pathways.. J Leukoc Biol.

[11] Arthos J, Rubbert A, Rabin RL, Cicala C, Machado E (2000). CCR5 signal transduction in macrophages by human immunodeficiency virus and simian immunodeficiency virus envelopes.. J Virol.

[12] Kohler JJ, Tuttle DL, Coberley CR, Sleasman JW, Goodenow MM (2003). Human immunodeficiency virus type 1 (HIV-1) induces activation of multiple STATs in CD4+ cells of lymphocyte or monocyte/macrophage lineages.. J Leukoc Biol.

[13] Coberley CR, Kohler JJ, Brown JN, Oshier JT, Baker HV (2004). Impact on genetic networks in human macrophages by a CCR5 strain of human immunodeficiency virus type 1.. J Virol.

[14] Tomkowicz B, Lee C, Ravyn V, Cheung R, Ptasznik A (2006). The Src kinase Lyn is required for CCR5 signaling in response to MIP-1beta and R5 HIV-1 gp120 in human macrophages.. Blood.

[15] Guntermann C, Murphy BJ, Zheng R, Qureshi A, Eagles PA (1999). Human immunodeficiency virus-1 infection requires pertussis toxin sensitive G-protein-coupled signalling and mediates cAMP downregulation.. Biochem Biophys Res Commun.

[16] Davis CB, Dikic I, Unutmaz D, Hill CM, Arthos J (1997). Signal transduction due to HIV-1 envelope interactions with chemokine receptors CXCR4 or CCR5.. J Exp Med.

[17] Geleziunas R, Xu W, Takeda K, Ichijo H, Greene WC (2001). HIV-1 Nef inhibits ASK1-dependent death signalling providing a potential mechanism for protecting the infected host cell.. Nature.

[18] Meier A, Alter G, Frahm N, Sidhu H, Li B (2007). MyD88-dependent immune activation mediated by human immunodeficiency virus type 1-encoded Toll-like receptor ligands.. J Virol.

[19] Beignon AS, McKenna K, Skoberne M, Manches O, DaSilva I (2005). Endocytosis of HIV-1 activates plasmacytoid dendritic cells via Toll-like receptor-viral RNA interactions.. J Clin Invest.

[20] Alfano M, Poli G (2005). Role of cytokines and chemokines in the regulation of innate immunity and HIV infection.. Mol Immunol.

[21] Hiscott J (2007). Convergence of the NF-kappaB and IRF pathways in the regulation of the innate antiviral response.. Cytokine Growth Factor Rev.

[22] Hiscott J, Nguyen TL, Arguello M, Nakhaei P, Paz S (2006). Manipulation of the nuclear factor-kappaB pathway and the innate immune response by viruses.. Oncogene.

[23] Lee SK, Park SO, Joe CO, Kim YS (2007). Interaction of HCV core protein with 14-3-3epsilon protein releases Bax to activate apoptosis.. Biochem Biophys Res Commun.

[24] Wang X, Grammatikakis N, Siganou A, Stevenson MA, Calderwood SK (2004). Interactions between extracellular signal-regulated protein kinase 1, 14-3-3epsilon, and heat shock factor 1 during stress.. J Biol Chem.

[25] Gu YM, Jin YH, Choi JK, Baek KH, Yeo CY (2006). Protein kinase A phosphorylates and regulates dimerization of 14-3-3 epsilon.. FEBS Lett.

[26] Ortutay C, Siermala M, Vihinen M (2007). Molecular characterization of the immune system: emergence of proteins, processes, and domains.. Immunogenetics.

[27] Aderem A, Smith KD (2004). A systems approach to dissecting immunity and inflammation.. Semin Immunol.

[28] Calvano SE, Xiao W, Richards DR, Felciano RM, Baker HV (2005). A network-based analysis of systemic inflammation in humans.. Nature.

[29] Cobb JP, Mindrinos MN, Miller-Graziano C, Calvano SE, Baker HV (2005). Application of genome-wide expression analysis to human health and disease.. Proc Natl Acad Sci U S A.

[30] Martinez FO, Sica A, Mantovani A, Locati M (2008). Macrophage activation and polarization.. Front Biosci.

[31] Hamilton TA, Burke B, Lewis CE (2002). Molecular basis of macrophage activation: from gene expression to phenotypic diversity.. The Macrophage.

[32] Adams DO, Hamilton TA (1984). The cell biology of macrophage activation.. Annu Rev Immunol.

[33] Baur A (2004). Functions of the HIV-1 Nef protein.. Curr Drug Targets Immune Endocr Metabol Disord.

[34] Olivetta E, Federico M (2006). HIV-1 Nef protects human-monocyte-derived macrophages from HIV-1-induced apoptosis.. Exp Cell Res.

[35] Freedman BD (2006). Mechanisms of calcium signaling and function in lymphocytes.. Crit Rev Immunol.

[36] Gougeon ML (2003). Apoptosis as an HIV strategy to escape immune attack.. Nat Rev Immunol.

[37] Jacque JM, Mann A, Enslen H, Sharova N, Brichacek B (1998). Modulation of HIV-1 infectivity by MAPK, a virion-associated kinase.. EMBO J.

[38] Popik W, Pitha PM (2000). Exploitation of cellular signaling by HIV-1: unwelcome guests with master keys that signal their entry.. Virology.

[39] Aquaro S, Bagnarelli P, Guenci T, De Luca A, Clementi M (2002). Long-term survival and virus production in human primary macrophages infected by human immunodeficiency virus.. J Med Virol.

[40] Goodenow MM, Rose SL, Tuttle DL, Sleasman JW (2003). HIV-1 fitness and macrophages.. J Leukoc Biol.

[41] Muthumani K, Wadsworth SA, Dayes NS, Hwang DS, Choo AY (2004). Suppression of HIV-1 viral replication and cellular pathogenesis by a novel p38/JNK kinase inhibitor.. AIDS.

[42] Popik W, Pitha PM (1998). Early activation of mitogen-activated protein kinase kinase, extracellular signal-regulated kinase, p38 mitogen-activated protein kinase, and c-Jun N-terminal kinase in response to binding of simian immunodeficiency virus to Jurkat T cells expressing CCR5 receptor.. Virology.

[43] Shapiro L, Heidenreich KA, Meintzer MK, Dinarello CA (1998). Role of p38 mitogen-activated protein kinase in HIV type 1 production in vitro.. Proc Natl Acad Sci U S A.

[44] Bukrinsky MI, Haggerty S, Dempsey MP, Sharova N, Adzhubel A (1993). A nuclear localization signal within HIV-1 matrix protein that governs infection of non-dividing cells.. Nature.

[45] Heinzinger NK, Bukinsky MI, Haggerty SA, Ragland AM, Kewalramani V (1994). The Vpr protein of human immunodeficiency virus type 1 influences nuclear localization of viral nucleic acids in nondividing host cells.. Proc Natl Acad Sci U S A.

[46] Wang X, Lewis DE (2001). CD86 expression correlates with amounts of HIV produced by macrophages in vitro.. J Leukoc Biol.

[47] Schuitemaker H, Kootstra NA, Fouchier RA, Hooibrink B, Miedema F (1994). Productive HIV-1 infection of macrophages restricted to the cell fraction with proliferative capacity.. EMBO J.

[48] Vazquez N, Greenwell-Wild T, Marinos NJ, Swaim WD, Nares S (2005). Human immunodeficiency virus type 1-induced macrophage gene expression includes the p21 gene, a target for viral regulation.. J Virol.

[49] Parish CR (1999). Fluorescent dyes for lymphocyte migration and proliferation studies.. Immunol Cell Biol.

[50] Mata-Haro V, Cekic C, Martin M, Chilton PM, Casella CR (2007). The vaccine adjuvant monophosphoryl lipid A as a TRIF-biased agonist of TLR4.. Science.

[51] Lleonart R, Naf D, Browning H, Weissmann C (1990). A novel, quantitative bioassay for type I interferon using a recombinant indicator cell line.. Biotechnology (N Y).

[52] Franchin G, Zybarth G, Dai WW, Dubrovsky L, Reiling N (2000). Lipopolysaccharide inhibits HIV-1 infection of monocyte- derived macrophages through direct and sustained down-regulation of CC chemokine receptor 5.. J Immunol.

[53] Verani A, Scarlatti G, Comar M, Tresoldi E, Polo S (1997). C-C chemokines released by lipopolysaccharide (LPS)-stimulated human macrophages suppress HIV-1 infection in both macrophages and T cells.. J Exp Med.

[54] Verani A, Sironi F, Siccardi AG, Lusso P, Vercelli D (2002). Inhibition of CXCR4-tropic HIV-1 infection by lipopolysaccharide: evidence of different mechanisms in macrophages and T lymphocytes.. J Immunol.

[55] Zybarth G, Reiling N, Schmidtmayerova H, Sherry B, Bukrinsky M (1999). Activation-induced resistance of human macrophages to HIV-1 infection in vitro.. J Immunol.

[56] Cicala C, Arthos J, Selig SM, Dennis G, Hosack DA (2002). HIV envelope induces a cascade of cell signals in non-proliferating target cells that favor virus replication.. Proc Natl Acad Sci U S A.

[57] Bennasser Y, Badou A, Tkaczuk J, Bahraoui E (2002). Signaling pathways triggered by HIV-1 Tat in human monocytes to induce TNF-alpha.. Virology.

[58] Badou A, Bennasser Y, Moreau M, Leclerc C, Benkirane M (2000). Tat protein of human immunodeficiency virus type 1 induces interleukin-10 in human peripheral blood monocytes: implication of protein kinase C-dependent pathway.. J Virol.

[59] Percario Z, Olivetta E, Fiorucci G, Mangino G, Peretti S (2003). Human immunodeficiency virus type 1 (HIV-1) Nef activates STAT3 in primary human monocyte/macrophages through the release of soluble factors: involvement of Nef domains interacting with the cell endocytotic machinery.. J Leukoc Biol.

[60] Janket ML, Manickam P, Majumder B, Thotala D, Wagner M (2004). Differential regulation of host cellular genes by HIV-1 viral protein R (Vpr): cDNA microarray analysis using isogenic virus.. Biochem Biophys Res Commun.

[61] Kootstra NA, Schuitemaker H (1998). Proliferation-dependent replication in primary macrophages of macrophage-tropic HIV type 1 variants.. AIDS Res Hum Retroviruses.

[62] DeFilippis VR (2007). Induction and evasion of the type I interferon response by cytomegaloviruses.. Adv Exp Med Biol.

[63] Weber F, Kochs G, Haller O (2004). Inverse interference: how viruses fight the interferon system.. Viral Immunol.

[64] Haller O, Kochs G, Weber F (2007). Interferon, Mx, and viral countermeasures.. Cytokine Growth Factor Rev.

[65] Hunt PW (2007). Role of immune activation in HIV pathogenesis.. Curr HIV/AIDS Rep.

[66] Gorry PR, Churchill M, Crowe SM, Cunningham AL, Gabuzda D (2005). Pathogenesis of macrophage tropic HIV-1.. Curr HIV Res.

[67] Kovarik P, Stoiber D, Novy M, Decker T (1998). Stat1 combines signals derived from IFN-gamma and LPS receptors during macrophage activation.. EMBO J.

[68] Kitaya K, Yasuo T, Yamaguchi T, Fushiki S, Honjo H (2007). Genes regulated by interferon-gamma in human uterine microvascular endothelial cells.. Int J Mol Med.

[69] Douek D (2007). HIV disease progression: immune activation, microbes, and a leaky gut.. Top HIV Med.

[70] Lester RT, Yao XD, Ball TB, McKinnon LR, Kaul R (2008). Toll-like receptor expression and responsiveness are increased in viraemic HIV-1 infection.. AIDS.

[71] Goodenow MM, Collman RG (2006). HIV-1 coreceptor preference is distinct from target cell tropism: a dual-parameter nomenclature to define viral phenotypes.. J Leukoc Biol.

[72] Ghaffari G, Tuttle DL, Briggs D, Burkhardt BR, Bhatt D (2005). Complex determinants in human immunodeficiency virus type 1 envelope gp120 mediate CXCR4-dependent infection of macrophages.. J Virol.

[73] Salemi M, Burkhardt BR, Gray RR, Ghaffari G, Sleasman JW (2007). Phylodynamics of HIV-1 in lymphoid and non-lymphoid tissues reveals a central role for the thymus in emergence of CXCR4-using quasispecies.. PLoS ONE.

[74] Tuttle DL, Harrison JK, Anders C, Sleasman JW, Goodenow MM (1998). Expression of CCR5 increases during monocyte differentiation and directly mediates macrophage susceptibility to infection by human immunodeficiency virus type 1.. J Virol.

[75] Tuttle DL, Anders CB, Aquino-De Jesus MJ, Poole PP, Lamers SL (2002). Increased replication of non-syncytium-inducing HIV type 1 isolates in monocyte-derived macrophages is linked to advanced disease in infected children.. AIDS Res Hum Retroviruses.

[76] Theodore TS, Englund G, Buckler-White A, Buckler CE, Martin MA (1996). Construction and characterization of a stable full-length macrophage-tropic HIV type 1 molecular clone that directs the production of high titers of progeny virions.. AIDS Res Hum Retroviruses.

[77] Gendelman HE, Orenstein JM, Martin MA, Ferrua C, Mitra R (1988). Efficient isolation and propagation of human immunodeficiency virus on recombinant colony-stimulating factor 1-treated monocytes.. J Exp Med.

[78] Ghaffari G, Passalacqua DJ, Bender BS, Briggs DJ, Goodenow MM (2001). Human lymphocyte proliferation responses following primary immunization with rabies vaccine as neoantigen.. Clin Diagn Lab Immunol.

